# HOMA-IR Index and Pediatric Psoriasis Severity—A Retrospective Observational Study

**DOI:** 10.3390/life14060700

**Published:** 2024-05-29

**Authors:** Adelina Maria Sendrea, Denis Iorga, Mihai Dascalu, Alina Suru, Carmen Maria Salavastru

**Affiliations:** 1 Pediatric Dermatology Department, Carol Davila University of Medicine and Pharmacy, 8 Eroilor Sanitari Boulevard, 050474 Bucharest, Romania; suru.pirvu@drd.umfcd.ro (A.S.); carmen.salavastru@umfcd.ro (C.M.S.); 2 Pediatric Dermatology Department, Colentina Clinical Hospital, 19-21 Stefan cel Mare Street, 020125 Bucharest, Romania; 3 Dermatology Research Unit, Colentina Clinical Hospital, 19-21 Stefan cel Mare Street, 020125 Bucharest, Romania; 4 Computer Science & Engineering Department, National University of Science and Technology Politehnica Bucharest, 313 Splaiul Independentei, 060042 Bucharest, Romania; denis_nicolae.iorga@upb.ro (D.I.); mihai.dascalu@upb.ro (M.D.); 5 Academy of Romanian Scientists, 3 Ilfov Street, 050044 Bucharest, Romania

**Keywords:** psoriasis, pediatric, insulin resistance, HOMA-IR, PASI, nail pitting

## Abstract

Psoriasis is a chronic inflammatory disease with specific cutaneous and nail lesions. Recent data has emphasized its systemic nature, highlighting metabolic conditions found in patients. Insulin resistance was identified in adult psoriasis, sometimes related to psoriasis severity. Data regarding this relationship in children are limited. Consequently, we tested the association between the Homeostatic Model Assessment for Insulin Resistance (HOMA-IR) and Psoriasis Area and Severity Index (PASI) using a retrospective dataset of 43 children with various types of psoriasis. First, we attempted to replicate the relationship between the HOMA-IR and PASI. Second, we explored potential associations between these variables and others in the dataset. The results illustrated no association between HOMA-IR and PASI (*p*-value = 0.512). The exploratory findings hinted at a connection between nail pitting and insulin resistance (*p*-value = 0.038), yet Bonferroni adjustments suggested the risk of a false-positive finding. Noteworthy associations were found between the HOMA-IR and body mass index (BMI) (*p*-value = 0.001), the PASI and quality of life impairment (*p*-value = 0.005), and psoriasis severity and type (*p*-value = 0.001). The null hypothesis that insulin resistance in children is not positively associated with psoriasis severity cannot be rejected. Pilot estimates of variables and covariates of interest are provided for further confirmatory studies assessing this hypothesis.

## 1. Introduction

Psoriasis is a chronic, relapsing inflammatory immune-mediated disease affecting up to 3.5% of the global population [1] with a variable geographic prevalence (from 0.5% in Asia to 8% in Norway) [2]. In Romania, epidemiological data regarding psoriasis are scarce. However, an epidemiological study identified an overall prevalence of 5.18% of this disease among Romanian patients, but with a heterogenous distribution among various regions of the country [3]. Clinically, it presents with specific cutaneous and nail lesions, and it can affect any age group, being more common in adults than in children; however, psoriasis has a childhood onset in approximately one-third of cases [1]. Psoriasis presents with various clinical types—the most common being psoriasis vulgaris (or chronic plaque psoriasis)—and severities, as determined by the Psoriasis Area and Severity Index (PASI). It can be associated with significant quality-of-life impairment regardless of the patient’s age, which can be assessed through a specific validated questionnaire—the Dermatology Life Quality Index (DLQI)—and its children’s version—the Children’s Dermatology Life Quality Index (CDLQI)—for patients younger than 16 years old.

Although initially considered to be strictly a skin-limited condition, in the past decades, psoriasis has been characterized more and more as a systemic inflammatory disease with various associated conditions, usually correlated with disease severity and duration but also with the patient’s age [4]. Aside from psoriatic onychopathy and arthritis, which represent well-acknowledged beyond-the-skin manifestations in psoriasis, various comorbidities have been identified, with special emphasis on cardiovascular and metabolic disorders, mainly in adult patients. However, evidence is also emerging regarding the association of such conditions with the pediatric population of patients. Among the most commonly identified so far in both adults and children are obesity, cardiovascular disease, metabolic syndrome, and insulin resistance [5,6].

Insulin resistance is a key factor involved in the pathogenesis of metabolic syndrome, along with obesity and perpetual low-grade inflammation [7]. Metabolic syndrome seems to be more frequently encountered in psoriasis patients than in the general population, with psoriasis severity linked in a directly proportional manner to metabolic syndrome in such patients [6]. Consequently, insulin resistance and psoriasis severity are expected to be associated. This is further supported by the fact that psoriasis and insulin resistance share some common pathophysiological factors, such as chronic inflammation and a genetic background. Both conditions exhibit overlapping inflammatory cytokines, including tumor necrosis factor-alpha (TNF-α) and interleukin 6 (IL-6), as well as leptin and adiponectin, which play crucial roles in their pathogenesis [6]. Moreover, other molecules and receptors have been proven to be involved in both psoriasis and insulin resistance (e.g., the glucagon-like peptide 1 receptor and incretin) [8].

Insulin resistance represents a pathological condition characterized by a reduced tissue reaction to insulin-induced cellular processes [9], and it is known to be one of the fundamental elements contributing to the development of other metabolic disorders, such as obesity, diabetes mellitus, arterial hypertension, or nonalcohol fatty liver disease [10]. There are various methods available for insulin resistance assessment, of which the Homeostatic Model Assessment for Insulin Resistance (HOMA-IR) index is widely used, having the advantage of being minimally invasive and based on a mathematical formula between fasting glucose and insulin serum levels. The cut-off value that indicates an elevated HOMA-IR index in adult populations is considered to be >2.5 [11], while in pediatric populations, such a value is not exactly established; some studies use variable pre-established fixed values (between 1.8 and 3.16), while others use age- and sex-related percentiles [12,13].

There are limited data on pediatric psoriasis patients regarding insulin resistance and metabolic syndrome associations. However, the prevalence of insulin resistance in children with psoriasis is believed to be almost twice as frequent compared to non-psoriatic children, as some studies have identified prevalence ratios varying from 1.97 to 2.01 [5]. Moreover, in a prospective, single-center study on 60 pre-pubertal children with psoriasis, approximately one-third presented with elevated HOMA-IR index values, corresponding to an insulin resistance risk [13]. Additionally, in a case–control study performed on 44 pediatric patients with psoriasis, it was identified that such patients exhibit elevated HOMA-IR values compared to healthy controls, with the most accurate indicator of insulin resistance being the body mass index (BMI) [14]. In the Romanian population, data regarding these associations in children with psoriasis are missing.

Based on the evidence presented so far, this study aims to evaluate the HOMA-IR index among pediatric patients with various severity degrees of psoriasis aged >6 years old, who attended a tertiary care referral center in Bucharest, Romania.

## 2. Materials and Methods

We performed a retrospective observational study on pediatric patients who were referred to Colentina Clinical Hospital—Pediatric Dermatology Department, Bucharest, Romania, East-Europe, for one year (from January 2023 to January 2024) and who complied with the following inclusion criteria: age between 6 and 17 years old and clinical diagnosis of psoriasis. Children aged <6 years old with other dermatological diagnoses than psoriasis, and psoriasis patients with a pre-diagnosed metabolic personal medical history or who were previously treated with both topical and systemic agents, were excluded. Given the inclusion and exclusion criteria and using the consecutive sampling method, based on the electronic medical records, 43 patients were included in the analysis. Clinical data (age, sex, height, mass, psoriasis type, nail involvement—pitting and oil spots—PASI scores, DLQI and CDLQI questionnaires), family history (dermatologic and metabolic), and paraclinical parameters (fasting serum glucose and insulin, erythrocyte sedimentation rate—ESR) were registered for all participants. Biomarkers (e.g., triglycerides—TG, high density lipoprotein cholesterol—HDL-C) were also registered for several patients.

The study comprised two phases. In the first phase, we sought to analyze the potential relationship between psoriasis severity and insulin resistance in pediatric patients. In the second phase, we sought to generate new hypotheses regarding the two variables of interest and provide estimates of covariates for future confirmatory studies assessing the same relationship. All the statistical analyses were performed using R version 4.2.3 (2023-03-15) software.

### 2.1. Replication of HOMA-IR and PASI Association

The study’s first phase involved testing the positive association between insulin resistance and psoriasis severity in our cohort. Insulin resistance was measured through the HOMA-IR index. Psoriasis severity was evaluated using the PASI.

The HOMA-IR index was calculated for each patient using the following formula:

$$\frac{\left[Fasting\;insulin\;\left(\frac{\mu U}{mL}\right)\times Fasting\;glucose\;\left(\frac{mg}{dL}\right)\right]}{405}$$

The cut-off value was established at 2.5, with cases being subdivided into normal (<2.5) and elevated (≥2.5) HOMA-IR indexes. 

Based on the PASI score, cases were subdivided into mild (PASI 0–5), moderate (PASI 6–10), and severe (PASI > 11) psoriasis. 

Inferential procedures were conducted on both the initial variables (i.e., the PASI and HOMA-IR) and their transformed variants (i.e., psoriasis severity which was classified as mild/moderate/severe and HOMA-IR levels which were defined as normal/elevated). The decision to use both the initial and transformed variables was taken in order to observe if the relationship between the variables changed when using the recoded categories. Non-parametric tests were considered appropriate given the non-normal distributions of HOMA-IR (Shapiro–Wilk *p*-value = 0.002) and PASI (Shapiro–Wilk *p*-value = 0.001) variables.

A one-tailed Kendall’s rank correlation procedure was first conducted using the PASI score and HOMA-IR index values. Furthermore, a one-tailed Cochran–Armitage test procedure was conducted on the cross-table between HOMA-IR index levels (normal/elevated) and psoriasis severity (mild/moderate/severe). This latter procedure is designed to test for an alternative hypothesis of a trend (i.e., a trend in the proportion of HOMA-IR levels across the levels of psoriasis severity). The one-tail variants were opted for, as a positive association between HOMA-IR–HOMA-IR levels and PASI–psoriasis severity was expected. A *p*-value of 0.05 was selected as the significance threshold. The Bonferroni correction was considered in interpreting the results, given that the null hypothesis would have been rejected if either of the two tests were significant [15].

### 2.2. Analysis of Bi-Variate Relationships

The second phase of the study involved exploring bi-variate relationships between the HOMA-IR index–PASI score and other variables collected from the medical records of pediatric patients, as listed in Table 1. The aim of this phase was to detect signals pointing to potential novel hypotheses regarding the two variables of interest. Both the initial variables (i.e., PASI and HOMA-IR index values) and their transformed variants (i.e., psoriasis severity which classified as mild/moderate/severe and HOMA-IR index levels which were defined as normal/elevated) were used.

As regards the other variables used in the exploratory phase, the Quantitative Insulin Sensitivity Check Index (QUICKI) was measured using the following formula:

$$\frac{1}{\log\left[fasting\;insulin\;\left(\frac{uU}{mL}\right)\right]+\log\left[fasting\;glucose\left(\frac{mg}{dL}\right)\right]}$$

The cut-off value for the QUICKI index was established at 0.34, and cases were further subdivided into low QUICKI levels (<0.349), correspondent to insulin resistance risk, and normal QUICKI levels (>0.35) [16]. 

The presence of inflammatory syndrome was decided based on elevated values for the ESR. The family history variables were assessed through anamnesis. Moreover, the metabolic family history was assessed based on the existence of obesity, diabetes mellitus, dyslipidemia, or arterial hypertension among first-degree family members. 

Based on the mass in kilograms (kg) and height in centimeters (cm), the BMI and the corresponding age and sex percentiles (pctl) were determined using the CDC BMI Percentile Calculator for Child and Teen for children older than 2 years [17] and the Ped(z) Pediatric Calculator with CDC/WHO data [18] for children younger than 2 years; in the next step, based on the resulting percentiles, study participants were divided according to their nutritional status as underweight (pctl < 5), normal weight (pctl = 5–84), overweight (pctl = 85–94), or obese (pctl ≥ 95). 

The psoriasis type was determined by a thorough clinical examination performed by dermatologists with experience in pediatric dermatology, identifying in our cohort four types of psoriasis: vulgaris, inverse, guttate, and follicular. Quality-of-life impairment was evaluated using the DLQI and CDLQI for patients aged <16 years old. Further, based on DLQI or CDLQI values determining the quality-of-life effect of psoriasis, patients were grouped into two subgroups: small effect and no effect (DLQI = 0–6) and moderate and large effect (DLQI > 6), respectively [19]. Furthermore, serum trglycerides (TGs) and high-density lipoproteins cholesterol (HDL-C) were assessed, both expressed in milligrams/deciliter (mg/dL), and a TG/HDL-C ratio was performed.

It should also be noted that two cases involving the psoriasis type (i.e., guttate and follicular) were removed from the analyses. Additionally, one case involving nutritional status (i.e., underweight) was removed from the analysis. The decision to remove these cases was made as the number of patients in the aforementioned categories was low, thus not allowing meaningful comparisons in our cohort. Moreover, 14 cases were removed from the analyses involving TG and 15 cases from the analyses involving the HDL-C and TG/HDL-C ratios due to missing data. No cases were removed in the other procedures.

Inferential tests using categorical variables were conducted using the Pearson chi-square (when testing for HOMA-IR index levels) and the two-tailed Cochran–Armitage tests (when testing for psoriasis severity levels). Tests involving correlations between two quantitative variables or two ordinal variables were conducted using the two-tailed Kendall’s rank correlation procedure. Tests involving a quantitative and a categorical variable were performed either using the two-tailed Wilcoxon–Mann–Whitney procedure (when the categorical variable was dichotomous) or the two-tailed Jonckheere–Terpstra procedure (when the categorical variable was ordinal). 

A 0.05 level of significance was taken as a reference for identifying noteworthy associations. Exact *p*-values were reported. A Bonferroni type I error adjustment was also used to interpret the results. The correction was deemed appropriate for tests involving the same null hypothesis, where at least one significant result would have been required to reject it [15]. More precisely, the level of significance was adjusted for multiple tests analyzing both initial and transformed variables, as well as for procedures related to the same null hypothesis (i.e., there is no relationship between insulin resistance and psoriasis). Kendall’s tau value was used to report the effect size for significant relationships, except for the Wilcoxon–Mann–Whitney procedure, where the r value was used. Confidence intervals (C.I.s) were computed for the correlation coefficient–effect size value in cases of noteworthy associations.

## 3. Results

Fasting glucose levels showed a mean = 85.29, median = 84.90, and a standard deviation (SD) of 7.24; while fasting insulin levels presented with a mean = 15.39, median = 14.9, and SD = 7.13. The resulting variable (HOMA-IR index) had a mean of 3.93, a median of 3.2, and an SD of 1.99. Based on the established cut-off value (2.5), we identified n = 27 (62.8%) cases with an elevated HOMA-IR, while n = 16 (37.2%) presented with a normal HOMA-IR index. The PASI score had a mean of 5.18, a median of 3.8, and an SD of 4.69, identifying mostly mild cases (n = 26 (60.5%)), followed by moderate (n = 11 (25.5%)), and severe (n = 6 (14%)) ones. 

The study’s first phase involved an attempt to replicate the association between insulin resistance and psoriasis severity. Kendall’s rank correlation procedure between the HOMA-IR and the PASI score suggested the lack of a positive association between the two variables (tau = −0.003; *p*-value = 0.512). The same was indicated by the Cochran–Armitage test (*p*-value = 0.423) applied to the transformed variables.

The second phase of the study involved exploring bi-variate relationships between HOMA-IR–PASI scores and other variables collected from the medical records of the study participants. Table 2 highlights associations between the variables in the database and the HOMA-IR scores, while Table 3 illustrates similar information related to the PASI score.

As can be observed from Table 2, several noteworthy associations were identified based on the nominal *p*-values. The HOMA-IR and QUICKI were negatively correlated for both the initial (tau = −0.891; −0.994 C.I.95% −0.732; *p*-value = 0.000) and the transformed versions of the variables. The BMI and HOMA-IR values were positively correlated for both the initial (tau = 0.366; 0.145 C.I.95% 0.565; *p*-value = 0.001) and the transformed (r effect size = 0.403; 0.125 C.I.95% 0.624; *p*-value = 0.008) version of HOMA-IR. In contrast, the association between the HOMA-IR and nutritional status almost attained the level of significance only when using the initial HOMA-IR scores (tau = 0.235; −0.032 C.I.95% 0.466; *p*-value = 0.057).

A relationship could also be observed between the initial HOMA-IR values and nail pitting, with those presenting signs of nail pitting having consistently higher HOMA-IR scores than those without (r effect size = −0.316; −0.575 C.I.95% −0.033; *p*-value = 0.038). However, this relationship was not detected using the recoded HOMA-IR levels (*p*-value = 0.159).

A positive association was also identified between blood insulin and the HOMA-IR using both the initial (tau = 0.821; 0.673 C.I.95% 0.934; *p*-value = 0.000) and the transformed variables. As regards the relationship between blood glucose and the HOMA-IR, a potential signal regarding their association was detected only for the initial variables (tau = 0.206; −0.017 C.I.95% 0.427; *p*-value = 0.052).

Another relationship highlighted in Table 2 refers to that between the HOMA-IR index and the presence of inflammatory syndrome, with those presenting with an elevated ESR having consistently higher HOMA-IR values than those without it (r effect size = −0.288; −0.555 C.I.95% 0.006; *p*-value = 0.058). Here again, the relationship was not detected when the normal/elevated levels of the HOMA-IR were used (*p*-value = 0.249).

Table 3 illustrates two noteworthy associations with the PASI score. First, pediatric patients with the vulgaris type of psoriasis presented consistently higher PASI values than those with the inverse type of psoriasis (r effect size = −0.611; −0.775 C.I.95% −0.384; *p*-value = 0.000). This relationship also holds when using the PASI score recoded into the three levels of severity (tau = 0.506; 0.267 C.I.95% 0.746; *p*-value = 0.001). 

Second, quality-of-life impairment positively correlated with the PASI score (tau = 0.303; 0.062 C.I.95% 0.517; *p*-value = 0.005), with the proportions of patients presenting moderate and severe quality-of-life impairment being highest among severe psoriasis cases and lowest among mild cases (tau = −0.402; −0.681 C.I.95% −0.122; *p* = 0.011).

Next, two Bonferroni corrections were applied to interpret the results. The first involved dividing the significance threshold by two for each test, given that each hypothesis was tested twice using both the initial and the transformed variables. This lowered the significance level from 0.05 to 0.025 for all the tests. The significance level was also lowered for tests focused on the same null hypothesis (i.e., no association between psoriasis and insulin resistance). This involved lowering the significance threshold from 0.025 to 0.002 for the following associations: psoriasis type and HOMA-IR, nail involvement and HOMA-IR, nail pitting and HOMA-IR, nail oil drop and HOMA-IR, family skin condition history and HOMA-IR, QUICKI and PASI, blood glucose and PASI, blood insulin and PASI, TG and PASI, HDL-C and PASI, and TG/HDL-C ratio and PASI.

The two correction procedures resulted in identifying the following relationships as potentially false positives: nail pitting and HOMA-IR, HOMA-IR and nutritional status, inflammatory syndrome and HOMA-IR, and blood glucose and HOMA-IR. Conversely, the Bonferroni corrections suggested the following relationships as true positives: HOMA-IR and QUICKI, HOMA-IR and BMI, HOMA-IR and blood insulin, PASI and psoriasis type, and PASI and quality-of-life impairment. These were also the relationships that were significant using both the initial and the transformed variables.

## 4. Discussion

In our study population, almost two-thirds of patients (62.8%) had an elevated value of the HOMA-IR index, corresponding to the risk of insulin resistance. However, considering that we used 2.5 as a cut-off value, instead of the other values or percentiles used in other pediatric studies [12,13], this percentage might be underestimated in our cohort of patients. Further research should be performed to establish a universally accepted cut-off value for children regarding insulin resistance evaluation through the HOMA-IR index.

Research regarding insulin resistance in children with psoriasis is scarce and controversial, as there are few data reports regarding this potential link. Most studies report data regarding the presence of metabolic syndrome (or its components, such as obesity and diabetes mellitus), not with a particular focus on insulin resistance itself in this subgroup of patients. A case–control study involving Portuguese patients with ages between 5 and 15 years old found a greater incidence of metabolic syndrome, but with no difference regarding insulin resistance presence, in children with moderate and severe chronic plaque psoriasis compared to healthy, non-psoriatic controls [20]. A different case–control study conducted in India examined psoriasis patients over the age of 15, including adults. The study found that these patients had notably elevated levels of serum fasting insulin and HOMA-IR index values, but no significant variations in serum fasting glucose levels when compared to individuals without psoriasis [21]. By comparison, in our research, the included patients had different age range (6–17 years old), and the study design was different, lacking a control group. One study on German children identified increased rates of various metabolic comorbidities (i.e., diabetes mellitus and obesity) in juvenile psoriasis, but without mentioning the HOMA-IR index [22]. Moreover, another research showed that, in comparison to atopic dermatitis cases, pediatric psoriasis patients exhibit notably higher rates of such comorbidities, but without specifically mentioning insulin resistance [23]. A systematic review and meta-analysis that comprised 17 studies (either case–control, cross-sectional, or cohort type) [24] identified significantly increased rates of various metabolic comorbidities among children diagnosed with psoriasis (e.g., obesity, diabetes, and metabolic syndrome). Data from a prospective study on prepubertal patients (ages between 3 and 10 years) with psoriasis identified that almost one third (27%) presented with increased HOMA-IR index values; the same patients also had increased prevalence rates of overweight/obesity status [13].

Our study failed to replicate the link between insulin resistance and psoriasis severity in pediatric patients. A potential link between these two variables has been identified in several studies involving adult psoriasis patients. Thereby, in various studies involving patients aged >18 years old with the psoriasis vulgaris type, a positive correlation between psoriasis severity and the HOMA-IR index was identified [6,25,26,27], with PASI values increasing the risk of HOMA-IR index elevation by 8.57 [25]. In our study, aside from evaluating pediatric patients, the study group comprised two types of psoriasis (involving inverse psoriasis, along with psoriasis vulgaris).

Nominal *p*-values computed in the exploratory phase of the current study suggested a potential relationship between nail pitting and insulin resistance. However, the Bonferroni correction suggested a considerable risk of a false-positive finding. The literature regarding this relationship is scarce. In a case–control study on adult Egyptian patients with psoriasis, a significant correlation between insulin resistance and psoriasis severity was identified, especially in patients with nail involvement, without specifically mentioning the type of nail lesion [28]. In another study involving adult patients with psoriasis, metabolic syndrome was identified with an increased prevalence in those with nail pitting [29]. Additionally, in a publication evaluating the spectrum of nail changes in diabetes mellitus patients, about 8.3% of such patients presented with nail pitting, having a previously diagnosis of psoriasis [30]. Hence, future studies with different, more suitable designs (i.e., case–control) and proper patient samples should consider exploring the potential link between insulin resistance and nail involvement in pediatric psoriasis patients.

Similarly, the nominal *p*-values suggested a potential connection between insulin resistance and inflammation (assessed through ESR). The ESR, although presenting with abnormal values in both psoriasis [31,32] and insulin resistance, does not seem to be very specific for these diseases, as various inflammation markers appear to be more correlated [32,33]. Furthermore, our study’s corrected level of significance suggested a potential false-positive finding. Consequently, future studies with other inflammation parameters (e.g., C3, high-sensitivity C-reactive protein) should be considered in exploring the link between insulin resistance and inflammation in psoriasis patients.

Other relationships identified in the present study were between HOMA-IR and QUICKI, HOMA-IR and blood insulin, HOMA-IR and BMI, PASI and psoriasis vulgaris clinical type, and PASI and DLQI/CDLQI, respectively. Taking into consideration that the HOMA-IR and QUICKI indexes are determined by different mathematical formulas that rely on the same parameters (i.e., fasting serum glucose and insulin), the relationship between them was expected. Similarly, given the fact that the HOMA-IR is an assessor of insulin resistance, its relationship with blood insulin levels is not surprising. Regarding the connection between the HOMA-IR and BMI, it is known that obesity and insulin resistance are significantly interlinked, since adipose tissue acts as a metabolically active endocrine organ, producing free fatty acids, reactive oxygen species, and various pro-inflammatory cytokines, all of which contribute to insulin resistance pathophysiological mechanisms [34,35]. Furthermore, the positive correlation between psoriasis severity and the vulgaris type of psoriasis, compared to inverse psoriasis, is not a surprise, as patients with vulgaris type tend to have a more extensive body surface area affected by lesions compared to those with inverse psoriasis, therefore resulting in higher PASI score values. Finally, the correlation between psoriasis severity (as measured by PASI) and quality-of-life impairment (as determined by DLQI/CDLQI) was also expected, taking into account that moderate/severe cases of psoriasis determine significant disturbances in various aspects of the patient’s quality of life, due to more extensive lesions and significantly more intense symptoms (such as pruritus, stinging, or a burning sensation on the skin); moreover, such patients might present with a poorer treatment response and treatment compliance compared to mild-severity cases.

The potential strengths of this study are represented by the thorough clinical assessment of the patients performed by dermatologists with experience in pediatric dermatology and the evaluation of other clinical types than psoriasis vulgaris in the sample (i.e., inverse psoriasis). The possible limitations are represented by the lack of a pre-data power-based sample size determination for the replication endeavor, a single-center study design, a potentially biased sample selection determined by the consecutive sampling method, the use of the conservative Bonferroni correction, and the lack of a substantive model involving potential cofounders, suppressors, or mediators of the relationship between the two variables of interest.

As a power-based sample size determination was not conducted before gathering the data, it is unclear whether the lack of association between the HOMA-IR and insulin resistance is due to a small sample size or a small effect size. In this sense, the upper confidence interval bound on the tau value of the correlation between the two variables is 0.281, suggesting, at most, a very weak true correlation coefficient. Likewise, regarding a potentially biased sample, the exploratory phase revealed that potential covariates of the HOMA-IR and PASI (e.g., psoriasis type, nail pitting) were relatively balanced in the sample. However, the missing data related to TGs, HDL, and the TG/HDL ratio prevent any definitive conclusion. Furthermore, the Bonferroni correction was reported in addition to the nominal *p*-values, thus allowing for alternative adjustment or interpretation approaches. Finally, concerning the lack of a substantive model involving potential co-founders, suppressors, or mediators of the relationship between the HOMA-IR and PASI, the current study represents a first step in this direction, the data being available on request for further studies. 

While the current study did not identify a relationship between insulin resistance and psoriasis in children, the aforementioned limitations suggest that, before more evidence is available in this direction, proper family and patient education regarding certain lifestyle modifications to prevent insulin resistance, diabetes, obesity, and metabolic syndrome development in children diagnosed with various clinical types and severities of psoriasis remain important.

## 5. Conclusions

To our knowledge, this is the first study to explore the potential connection between insulin resistance (as determined by the HOMA-IR index) and psoriasis in a Romanian cohort of pediatric patients. Although a significant proportion of the study population presented with HOMA-IR index values corresponding to insulin resistance, the study failed to identify a statistically significant connection between psoriasis severity (as evaluated by the PASI) and insulin resistance presence. Furthermore, while the evidence regarding the link between insulin resistance and nail pitting is relatively weak, this hypothesis should be further assessed in future studies.

Our findings highlight the need for further assessment of insulin resistance in pediatric psoriasis patients. Future studies with different designs (e.g., multi-centric and with an appropriate sampling size and random selection methods, or case–control type) are needed for a more accurate evaluation of the link between insulin resistance and various psoriasis parameters in the pediatric population. In this regard, the current study provides estimates of variables and covariates of interest. Physicians assessing and treating children with psoriasis (i.e., dermatologists, pediatricians, and endocrinologists) should be aware of this potential connection, to perform insulin resistance-related screening procedures in psoriasis children for a more integrated, multidisciplinary management of such patients.

## Figures and Tables

**Table 1:** Descriptive statistics for variables used in exploratory phase.

Variable	Details	Mean/Median/p1 *	SD/IQR/p2 *
HOMA-IR	Insulin resistance	*3.2*	*2.1*
HOMA-IR levels	Elevated/Normal	0.62	0.38
QUICKI	Insulin sensitivity	*0.3*	*0.03*
QUICKI levels	Low/Normal	0.70	0.30
PASI	Psoriasis severity	*3.8*	*4.8*
Psoriasis severity	Mild/Moderate/Severe	0.60	0.25/0.14
Psoriasis type	Inverse/Vulgaris (2 removed cases)	0.46	0.54
Age	In years	12.02	3.43
Age category	Adolescence/Middle childhood	0.60	0.40
Sex	Female/Male	0.65	0.35
Nail involvement	No/Yes	0.48	0.52
Quality-of-life impairment	Children’s Dermatology Life Quality Index	*5.0*	*7.5*
Quality-of-life imp. category	Moderate and Large (>6)/Small and No effect (0–6)	0.39	0.61
Nail pitting	No/Yes	0.58	0.42
Nail oil spot	No/Yes	0.79	0.31
BMI	Body mass index	23.63	5.21
Nutritional status	Normal weight/Overweight/Obese (1 removed case)	0.38	0.19/0.43
Family skin condition history	No/Yes	0.70	0.30
Metabolic family history	No/Yes	0.79	0.21
Inflammatory syndrome	No/Yes	0.67	0.33
Blood glucose	mg/dL	85.29	7.24
Blood insulin	uU/mL	15.39	7.13
TG	mg/dL (14 missing data cases)	*79.0*	*47.3*
HDL-C	mg/dL (15 missing data cases)	*46.1*	*20.2*
TG/HDL-C ratio	Ratio of TG and HDL-C (15 missing data cases)	*1.6*	*1.34*

* p1 = proportion of first category; p2 = proportion of second and third categories; SD = standard deviation; IQR = interquartile range; HOMA-IR = Homeostatic Model Assessment for Insulin Resistance; QUICKI = Quantitative Insulin Sensitivity Check Index; PASI = Psoriasis Area and Severity Index; BMI = body mass index; TG = triglycerides; HDL-C = high density lipoprotein-cholesterol; median and IQR are computed for values with italics and one decimal (except for QUICKI, where two decimals were used).

**Table 2:** Results of significance tests between HOMA-IR and other variables in dataset.

Variable of Interest	Variable in the Dataset	*p* Value
	QUICKI	0.000 ***
HOMA-IR values	Psoriasis type	0.266
	Age	0.267
	Sex	0.798
	Nail involvement	0.451
	Quality-of-life impairment	0.899
	Nail pitting	0.038 **
	Nail oil drop	0.632
	BMI	0.001 ***
	Nutritional status	0.057
	Family skin condition history	0.863
	Metabolic family history	0.131
	Inflammatory syndrome	0.058
	Blood glucose	0.052
	Blood insulin	0.000 ***
	TG	0.573
	HDL-C	0.205
	TG/HDL-C ratio	0.592
HOMA-IR levels (normal/elevated)	QUICKI levels	0.000 ***
	Psoriasis type	0.974
	Age category	0.910
	Sex	0.781
	Nail involvement	0.906
	Quality-of-life imp. categories	0.910
	Nail pitting	0.159
	Nail oil drop	0.372
	BMI	0.008 ***
	Nutritional status	0.330
	Family skin condition history	0.816
	Metabolic family history	0.372
	Inflammatory syndrome	0.249
	Blood glucose	0.182
	Blood insulin	0.000 ***
	TG	0.559
	HDL-C	0.230
	TG/HDL-C ratio	0.509

*** *p* < 0.01, ** *p* < 0.05.

**Table 3:** Results of significance tests between PASI and other variables in dataset.

Variable of Interest	Variable in the Dataset	*p* Value
PASI values	QUICKI	0.564
	Psoriasis type	0.000 ***
	Age	0.759
	Sex	0.583
	Nail involvement	0.388
	Quality-of-life impairment	0.005 ***
	Nail pitting	0.498
	Nail oil drop	0.085
	BMI	0.825
	Nutritional status	0.906
	Family skin condition history	0.346
	Metabolic family history	0.302
	Inflammatory syndrome	0.559
	Blood glucose	0.842
	Blood insulin	0.426
	TG	0.319
	HDL-C	0.858
	TG/HDL-C ratio	0.383
Psoriasis severity (mild/moderate/severe)	QUICKI levels	0.393
	Psoriasis type	0.001 ***
	Age category	0.697
	Sex	0.189
	Nail involvement	0.174
	Quality-of-life imp. categories	0.011 **
	Nail pitting	0.151
	Nail oil drop	0.100
	BMI	0.514
	Nutritional status	0.629
	Family skin condition history	0.663
	Metabolic family history	0.146
	Inflammatory syndrome	0.260
	Blood glucose	0.673
	Blood insulin	0.790
	TG	0.177
	HDL-C	0.809
	TG/HDL-C ratio	0.252

*** *p* < 0.01, ** *p* < 0.05.

## Data Availability

The original contributions presented in the study are included in the article; further inquiries can be directed to the corresponding author/s.
